# 

**Published:** 1891-06-20

**Authors:** 


### NOTICE TO CORRESPONDENTS.

All MS., Letters, Books for Review, and other matters intended for the Editor should be addressed The Editor, The Lodge, Porchester Square, London, W.

The Editor cannot undertake to return rejected M.S., even when accompanied by stamped directed envelope.

**Notice to Advertisers and Subscribers.**

All Advertisements, Orders for Copies of The Hospital, and Business Communications should be addressed to The Publisher, *not to the Editor*, The Hospital, 140, Strand, W.C.

The Cost of Subscription, post free, is as follows:—Per week, 1½d.; per quarter, 1s. 8d.; per half-year, 3s. 3d.; per annum, 6s. 6d. Foreign:—Per annum, 8s. 8d.; payable, in all cases, in advance.

**NOTICE.—The Index for Vol. IX., (ending March, 1891) is on sale at the Office of this Journal, price 2½d., post free.**

### General Charities.

[This Column is devoted to an account of work of charitable institutions other than Medical Charities. The Editor will be glad to receive reports or news of work at 140, Strand. *Philanthropy* should be plainly written on the envelope.]

Dr. Barnardo's twenty-fifth annual report, which is concise and interesting, tells a tale of much work and a great deal of success. The receipts by way of donation from all quarters during the past year amounted to £110,478, an increase of £3,755 over the year's receipts in 1889. Here is a contrast worth considering: In 1866 one little house was opened by Dr. Barnardo in Stepney Causeway, which gave shelter to waifs and strays whose only resting-place hitherto had been the common lodging-house, deserted markets, or any unobserved nook and corner. Now in 1891 "the Homes" number 41 several institutions, the age of the inmates varying from babies of three weeks old up to girls and boys of 17 and 18 years. A condensed summary of all receipts and payments is shown at the end of the report, the accuracy of which is vouched for by "Turquand, Youngs, and Co," consulting auditors.

It would take much more space than can be spared to give in detail all the numerous schemes of this vast organisation, but the first thing to be noticed is that no applicant is refused admission, "admit first, enquire afterwards" being Dr. Barnardo's principle. There are the General Offices at Stepney Causeway, with the Boys' Home, where the general age of the inmates is from 13 to 16 years, those older are sent to the Labour House for Destitute Youths; the younger ones are sheltered at Leopold House, at the Jersey Home, or the Children's Fold if between five and ten, and at Babies' Castle if below five years of age. Boys at the homes are taught on the "half-time" system, they are in school half the day, while the remaining hours are devoted to training in some technical handicraft, where our future bakers, blacksmiths, brushmakers, carpenters, printers, tailors, &c., may be seen fitting themselves for their future struggle for existence.

Forty-nine cottages and five larger households now compose the village of Barkingside, Ilford, where Dr. Barnardo in 1874 began his rescue of orphan and destitute girls. No barrack system is found in this model village, we are glad to note, for nothing can be more disastrous than that system of training, for in a vast community of children brought from the lowest depths of human degradation, vice is more certain to preponderate than virtue, and the individuality of the children is completely obliterated. Each cottage shelters about 20 girls, and at the head of each "family" there is a lady who is called "mother" by the inmates. School is attended, and training in domestic service given. After their training the girls are either emigrated or sent to domestic service- and the applications for their services are more numerous than the supply. There is a rescue home with a private address for young girls whose former associations necessitate a period of moral quarantine before they pass on to the village at Barkingside.

Open shelters for day and night at Stepney Causeway; a servants' registry and home for servants; a factory girls' institute; the Beehive Industrial Home and Laundry where girls who are too old for the home at Barkingside are trained, are some of the many departments of this institution. There is the Mission Church at Limehouse, which accommodates over 3,000 people, there is Her Majesty's Hospital with 75 beds, there is the convalescent home at Felixstowe, and a branch for the admission and special training of deaf mute and blind, crippled and deformed children. In point of fact, there is alleviation for every complaint and suffering, physical and mental, that the children waifs and strays are subject to. A farm school at Bromyard, in Worcestershire, given rent free by Mr. Richard Phipps, J.P., whose property it is, is a great addition to the work among the boys; the training received here fits them for work on the Industrial Farm of 10,000 acres in Manitoba. A new colonisation scheme is on foot by which boys who have been sent out four or five years ago by the Homes, and whose characters are good and who have saved 150 dollars, will be started as settlers each on a free homestead of a quarter section of Canadian Government land, and it is hoped that at the end of eighteen months the settler will be able to make his way without any further assistance from the Institutions. Dr. Barnardo is issuing a special appeal this year for funds to meet certain liabilities and to put the income of the Homes on a certain footing, and it is sincerely to be hoped that the money will be forthcoming. Here is an institution which has been doing excellent work for twenty-five years, and which can show practical results, which has to beg daily for funds, while money rolls in to the promoters of other schemes, who, because they blow their trumpets with grander and louder effect, are pronounced by a credulous public to be going to cure every evil under the sun. Dr. Barnardo is abused by many people becauses he announces everywhere that he is an evangelistic Protestant by principle, and that the principles of the Homes are the same. Well, for our part we are absolutely unsectarian. We are equally glad to help the work of every religion. Dr. Barnardo is a Protestant, and he is doing a splendid work, he is raising those whose ideas were dull and whose lives were useless to a consciousness of right and wrong, and to the knowledge that everybody must take his share of the world's work. Is not this enough for any of us? The happiness of 3,000 children's lives is worth the suppression of a little prejudice.

### Scraps and Gleanings.

The total sum collected by the Sleaford Hospital Saturday collection was £163 16s. 8d.

The collections at St. Jude's, South Kensington, on Hospital Sunday, amounted to £1,290 7s. 3d.

Mr. A. A. Soames has given £25, and Messrs. Reid and Co. £200, to the Hospital Sunday Fund.

A "Harrow" cot, as a companion to the "Eton" cot, has been established by old Harrovians in Ancoats Hospital.

During the recent epidemic of influenza in Chicago, immense quantities of phenacetine were prescribed, this seeming to be the favourite remedy.

During last month 77 tons of fish were seized at Billingsgate as being unfit for human food. The total weight of fish delivered was 13,183 tons.

Dr. E. Wagner has been appointed extern physician to the North Charitable Infirmary, Cork, and Dr. Richard Dalton has been appointed assistant extern physician.

The Trustees of Smith's Kensington Charity have sent £200 to the Hon. Alfred Lyttelton, Treasurer of the Children's Country Holidays Fund, 10, Buckingham Street, Strand.

Frank Chubb Ford, Esq., M.R.C.S., L.R.C.P. Lond., has been appointed house physician for six months to the City of London Hospital for Diseases of the Chest, Victoria Park.

The Hoxton branch of the Children's Happy Evenings Association was inaugurated last Friday by Lady Jeune. This makes the twentieth of the series of evening entertainments established by the Association.

The returns of the Registrar-General for the week ending March 6th state the deaths primarily attributed to influenza numbered 305, as compared with 266, 319, and 310 in the preceding three weeks.

It is reported at Stockholm, that Professor Rosander claims to have cured four cases of cancer by inoculation with some lymph of his own invention. Two of the cases were inoculated in the breast and two in the face.

The Friendly Trade Societies of Caversham and District held their second annual parade in aid of the funds of the Royal Berks Hospital on the 7th inst. The money collected, including church offertories, was £48 5s. 7½d.

The balance-sheet for 1890 of the Millar Hospital and Royal Kent Dispensary, shows that the income amounted to £7,561 3s., and the expenditure to £3,187 14s. 6d., leaving a balance at the bank of £4,373 9s. 6d.

After opening the new portions of All Saints' Convalescent Home at Eastbourne, on Saturday, the Prince and Princess of Wales will proceed to Upperton, and open the Children's Wards of the Princess Alice Memorial Hospital.

The sum of £25 has been handed by Miss Olga Garland and Mr. Harding Cox to the Hospital for Sick Children in Great Ormond Street, the proceeds, after paying expenses, of their matinée in aid of this institution, at Terry's, on the 23rd ult.

An alarming fire took place last Friday morning at the Home of the Sisters of St. Katherine, 32 and 33, Queen's Square, Bloomsbury. No. 33 was entirely gutted, but by dint of great exertions the chapel at the rear of No. 32 was saved from destruction.

The fourth festival dinner in aid of the funds of the Royal Alfred Aged Merchant Seamen's Institution took place at the Hotel Metropole on the 10th inst. Captain Sir James Anderson was in the chair, and subscriptions amounting to £4,107 were announced.

Miss Hester Russel, a student of the London School of Medicine for Women, has carried off the highest honours to be won in the M.B. examination of the Royal University of Ireland. Miss Russel held for six months the post of curator to the Royal Free Hospital.

Mr. Arthur Jackson has been unanimously elected Treasurer to the Sheffield Public Hospital in the place of the late Mr. Bernard Wake. As a memorial of Mr. Wake's great services the Board have resolved to start a convalescent fund for the assistance of patients leaving the hospital.

The Prince and Princess of Wales will visit St. Mary's Hospital, Paddington, at the end of July, to lay the foundation stone of an important and extensive block of buildings, about to be erected in Praed Street, completing the hospital in accordance with the original plan of the founders.

The second annual report of the Marlow Cottage Hospital states that 38 patients were admitted during last year. The income amounted to £364 2s. 0½d., being £141 10s. 9d. in excess of the expenditure. The committee have received about £200 towards the endowment fund, and have been promised £60 more.

The directors of the Edinburgh Sick Children's Hospital have resolved to build a new hospital. A suitable site has not yet been procured, but the directors of the Royal Infirmary are co-operating with the directors of the Children's Hospital, so it is hoped that an arrangement will soon be made.

A crowded mass meeting of property owners, ratepayers, and other inhabitants of Stamford Hill, Tottenham, was held last Saturday, for the purpose of protesting against the proposal of the Metropolitan Asylums Board to erect a fever hospital, capable of containing 500 beds, in St. Anne's Road, Stamford Hill.

The report for last year of the Iver, Langley, and Denham Cottage Hospital states that 70 patients were admitted. The year began with a favourable balance of £53 8s. 6d., and although various works have been carried out and extra expenses incurred, the year ended with a favourable balance of £26 7s. 4d., since increased by a donation of £14 15s.

At a meeting of the Metropolitan Asylums Board last Saturday, at which Sir E. Galsworthy presided, one of the managers complained of the size of the staff now there was so little to do; but it was explained that the authorities had to slightly anticipate events in the provision of staff, in order to be ready for patients when they arrived. The public would blame them much more if they were short of doctors and nurses at a critical time, than on account of a little additional expense in maintaining the ships in a state of preparedness.

### Books, &c., Received.

E. W. Allen.

"The Public Health: Leprosy and Vaccination." William Tebb. (Price 6d.).

Bailliere, Tindall, and Cox.

"Address on the Germ-Theory of Disease. With Specia lReference to Kochspiel." By D. Campbell Black, M.D. (Price 6d.)

"Journal de Medicine" (Paris).

"Etudes sur la Race." Dr. Lutaud. (Second Edition).

### The Student of Medicine.

[All communications for this Supplement should be addressed to The Editor of The Hospital, at 140, Strand, with the words, "Students' Column," written in the left hand top corner of the envelope.]

### EDITORIAL NOTES.

In a recent number of the *British Medical Journal* Professor Waller states his candid opinion concerning the unsatisfactory state of the first fellowship examination at the Royal College of Surgeons, and this he gives as the result of seven years' experience as a teacher at one of the large metropolitan medical schools. During this period he has carefully studied and compared the results of the passing and rejecting of candidates at this examination with those of the intermediate M.B.Lond., with the following conclusion: "That at the intermediate a sound man will probably pass, an unsound man will probably fail; while at the First Fellowship of the Royal College of Surgeons a sound or an unsound man may pass or fail, with very little margin of probability in favour of the former."

These opinions, which have just been published by a teacher in a London school, have for some years been generally acknowledged by professors and students of many of the leading universities, notably at Cambridge. We remember an occasion when as many as nine men went up from this university, some of them men who had a very good knowledge of their subjects, and they were every one ploughed. At this period it was considered fatal to go up for this examination from the university without first attending classes, of course, paying fees, to a school which supplied at least one of the examiners.

Professor Waller's letter is followed the next week by a communication from one of the rejected candidates at the recent examination, who signs himself "Gerechtikheit" (probably intended for Gerechtigkeit), and be points out that at the May examination three graduates of London at the recent B.Sc. have been rejected, though one of these graduates obtained the marks qualifying for the University Scholarship in physiology and another obtained honours, and he further adds: "That a physiologist placed so high in the stiffest physiology examination in Great Britain should be rejected at the Fellowship is nothing short of a scandal, for there can be no question here as to whether he knew his work. And in anatomy two demonstrators of anatomy at leading medical schools in London were similarly rejected in anatomy, to say nothing of other men well up in their work." "Gerechtikheit" then proceeds to compare the Fellowship examination with the London M.D., and suggests that for the Fellowship there is a premium on being coached in those medical schools where the examiners teach, and he says that in no examination is this better exemplified than in the late first Fellowship, for the hospital which supplied an examiner in anatomy and another in physiology got six men through out of six, whereas that which can train men to get the gold medal in the M.D. examination for the last four or five years consecutively has had three rejected out of three sent up.

"Gerechtikheit" is a graduate of the London University, and as such naturally "puffs" the London University examinations above all others. With most of his points, especially concerning the First Fellowship, we quite agree. He, however, makes an unfortunate remark when he refers to the gold medal for the London M.D., as we have heard it hinted by those who went up from other but the gold medal school par excellence, that this particular school had also provided half the examiners in the subject of medicine as well as the gold medalists, and the years in which students from this school have carried off the gold medal curiously correspond to those of the appointment of the examiner from the same school. This is, of course, but a mere coincidence. Thus from this point of view there is not much to be said in favour of either examination.

From what has come out, it is quite clear that not only is it necessary for a student who desires to get through the Fellowship, to attend classes and pay fees to the school which provides the greatest number of examiners for the time being, but also in order to run for the gold medal of the university he must do the same thing. We would not for one instant accuse the examiners of favouring their own men, but the fact nevertheless

THE STUDENT OF MEDICINE—(*continued*).

remains, and is worth investigating. The explanation is probably much as follows: Every man has some, often many, idiosyncrasies popularly known as "fads," both in his ideas and also in his teaching, and being in many cases a man of limited general ideas, he considers his "fads" as of momentary importance, and those of other men as idiotic. As long as what are termed the higher examinations are conducted on their present extremely narrow and unscientific principles, results such as have been stated must continue. How many of the examiners in anatomy for the first Fellowship are even devoting the chief part of their time to the subject they are examining in, and are not rather busy surgeons, whose knowledge of anatomy is only up to the recognised standard for operative work, and has been obtained in a more or less learning by heart or rule of thumb way, and not by any attempt at a scientific basis for their knowledge.

### APPOINTMENTS.

At St. Thomas's Hospital, E. C. Stubb, F.R.C.S., has been appointed resident surgeon. At the Blackburn and East Lancaster Infirmary, W. A. Bryant, M.B., C.M.Edin., has been appointed junior house surgeon. At the City of London Hospital for Diseases of the Chest, Victoria Park, Frank Chubb Ford, M.R.C.S., L.R.C.P., has been appointed house physician. At the Dunstable Friendly Benefit Societies' Medical Association, Charles John Lothbury, L.R.C.P.Lond., L.R.C.P., L.R.C.S.Edin., has been appointed resident medical officer. At the Eastern Dispensary, Bath, W. McDonogh Ellis, M.D.Brux., L.R.C.P. Lond., M.R.C.S., has been appointed honorary medical officer. At the Farringdon General Dispensary, Charles William Chapman, M.D.Durh., M.R.C.S.Lond., has been appointed honorary physician. At the General Hospital, Birmingham, Gilbert Barling, F.R.C.S.Eng., has been appointed honorary surgeon, and Thomas Sydney Short, M.B.Lond., D.P.H.Camb., M.R.C.S., L.S.A., assistant physician. At the Liverpool Royal Infirmary, J. Hill Abram, M.D., L.R.C.P.Lond., has been appointed pathologist. At the Mater Misericordiæ Hospital, Dublin, M.C.Staunton, M.B., &c., Royal University of Ireland, has been reappointed house surgeon. At the North Staffordshire Infirmary and Eye Hospital, Hartshill, W. H. Coupland has been appointed assistant house surgeon. At the Newcastle (New South Wales) Hospital, Henry Martin Doyle, M.R.C.P., L.R.C.S., L.S.A. Lond., has been appointed medical superintendent. At the Royal Infirmary, Liverpool, R. A. Bickersteth, M.B.Lond., M.R.C.S.Eng., has been appointed honorary assistant surgeon. At the Royal Bath Mineral Water Hospital, A. B. Brabazon, M.D.Aberd., L.R.C.S.I., has been appointed senior physician. At the Royal Westminster Ophthalmic Hospital, King William Street, C. G. MacLeod, M.B., C.M., has been appointed house surgeon. At the Royal Berks Hospital, Reading, Charles W. Marriott, M.D., M.R.C.S., has been appointed physician. At the Sussex County Hospital, Brighton, Edward Maynard, M.B., C.M., has been appointed house physician. At the Salford Union Infirmary, E. T. Larkham, M.R.C.S., L.R.C.P., has been appointed assistant medical officer. At the Warneford Hospital, Leamington Spa, George Dickinson, M.R.C.S., L.R.C.P., has been appointed house surgeon. Herbert Ward-Humphreys, L.R.C.P.Lond., M.R.C.S., appointed Surgeon to the Cheltenham Children's Hospital.

### VACANCIES.

The following vacancies are announced this week, the amount of salary in each case being given after the name of the institution:

**Resident Medical Officers.**

Bedford General Infirmary and Fever Hospital, Resident Surgeon—Salary £100, &c.
Bradford General Infirmary and Fever Hospital, Resident Surgeon—£100.
Dorset County Asylum, Assistant Medical Officer—Salary £120, &c.
East London Hospital for Children, Shadwell, E., House Surgeon.
General Hospital, Birmingham, Assistant House Surgeon.
General Infirmary at Gloucester and Gloucestershire Eye Institution, House Surgeon—Salary £100, &c.
General Infirmary, Leeds, Resident Casualty Officer—Salary £100, &c.; Resident Medical Officer—Salary £100, &c.
Horton Infirmary, Banbury, House Surgn. and Dispenser—Salary £60, &c.
Jessop Hospital for Women, Sheffield, House Surgeon—Salary £50, &c.
Liverpool Northern Hospital, Assistant House Surgeon—Salary £70, &c.
Lunatic Hospital, The Coppice, Nottingham, Assistant Medical Officer, Salary £100, &c.
North-West London Hospital, Kentish Town Road, N.W., Assistant Resident Medical Officer.
Parish of St. Leonard, Shoreditch, Clinical Assistant for Infirmary—Salary £40, &c.
Royal Free Hospital, Gray's Inn Road, Junior Resident Medical Officer.
Royal South Hants Infirmary, Southampton—Assistant to the House Surgeon.
Tunbridge Wells General Hospital, Res. House Surgeon—Salary £100.

### THE STUDENT OF MEDICINE—(*continued*).

Western General Dispensary, Marylebone Road, N.W., Second House Surgeon—Salary £50, &c.
West London Hospital, Hammersmith Road, House Physician and House Surgeon.
York County Hospital, Assistant House Surgeon—Salary £40, &c.

**Non-Resident Medical Officers.**

East London Hospital for Children, Shadwell, E., Assistant Physician.
General Hospital, Birmingham. Assistant Surgeon—Salary £100.
Guy's Hospital, S.E., five additional Assistant Dental Surgeons.
Queen's College, Birmingham, Medical Tutor and Demonstrator of Anatomy, also Demonstrator of Anatomy.
Royal Westminster Ophthalmic Hospital, King William Street, West Strand, Clinical Assistants.
St. Marylebone General Dispensary, Welbeck Street, W., Obstetric Physician.
University of Glasgow— Assistant Examiner in Physiology—Annual fee £30.
Victoria Hospital for Sick Children, Queen's Road, Chelsea, Assistant Physician to the out-patients : also Physician to the in-patients.
Victoria University, Holt Professorship of Physiology—Salary £375, plus share of students' fees.

### SCHOLARSHIPS AND PRIZES.

**Queen's College, Cork.**

In the Faculty of Medicine the following prizes have been awarded :—

Practical Anatomy (second year).—W. H. Garde, first; J. Reid, second. Third year : Wm. Scott, first; W. Rountree and Wm. McMath, equal.

Anatomy and Physiology (second year).—W. H. Garde, first. Third year : W. A. Rountree, first ; Wm. Scott, second.

Histology.—W. Rountree and W. A. Scott, equal second; Wm. McMath, third.

Practice of Medicine.—William Scott, first; William McMath, second.

Surgery.—William Scott, first.

Midwifery.—C. M. Corkery, first; W. C. Sullivan, second; D. O'Driscoll, third.

Materia Medica.—W. H. Garde, first; John Dundar, third.

Medical Jurisprudence.—D. J. Collins and P. T. O'Sullivan, equal first; C. Corkery and W. C. Sullivan, equal second.

Exhibition in Practical Medicine.—P. T. O'Sullivan.

**University College, London.**

The distribution of prizes by Sir Richard Quain took place on May 14th last. There was a representative gathering of past and present students, including Mr. Ericksen, F.R.S. (the President), Dr. Russell Reynolds, Professor Berkeley Hill, and Dr. John Williams (the Dean). The report was first read by the Dean, who stated that during the past year 346 students had attended classes of the medical faculty, including 86 new students. The following is a list of the prizes and medals :

Entrance Exhibitions—£100. F. W. Chandler, of Derby; £50 each (equal), J. H. Cook, of London, and S. E. Shoppee, of London. Atchinson Scholarship (£60 per annum for two years)—W. M. Stevens, of St. Ives. Atkinson Morley Scholarship (£45 per annum for three years)—G. F. Blacker, of Dublin. Bruce Medal—W. M. Stevens, of St. Ives. Cluff Memorial Prize (£15)—F. W. Wesley, of London.

Physiology.—Senior class, gold medal, E. F. Harwood, of Lisbon ; junior class, silver medal, J. H. Cook, of London.

Anatomy.—Senior class, gold medal, A. P. Nuttall, of Bury; silver, J. P. Kitson, of Southsea ; junior class, silver medal, T. H. C. Stevenson, of Ardcoen.

Medicine.—Gold medal, W. M. Stevens, of St. Ives; first silver, W. L. Andriezen, of Ceylon; second silver (equal), E. L. Hughes, of London, and H. M. Richards, of Cardiff.

Surgery.—Gold medal, W. M. Stevens, of St. Ives ; first silver, H. M. Richards, of Cardiff ; second silver, E. M. Smith, of Huntingdon.

Pathology.—Take Silver Medal, W. L. Andriezen, of Ceylon ; Take bronze medal, F. V. Bunch, of London.

Practical Surgery.—Erichsen prize, G. E. Newby, of London.

Clinical Medicine.—Senior class, Fellowes gold medal, W. M. Stevens, of St. Ives; Fellowes silver, H. R. Smith, of Putney; junior class, Fellowes silver medal (equal), F. V. Bunch, of London, and C. G. Spencer, of New Zealand.

Clinical Surgery.—Liston gold medal, W. L. Andriezen, of Ceylon.

Clinical Dental Surgery.—Silver medal, H. R. Smith, of Putney.

Sir Richard Quain, in the course of his address after the distribution, in very appropriate terms, congratulated the students who had gained prizes, and referred to half a century ago, when he was a student in the same hall, anxiously awaiting what might be in store for him.

### PASS LISTS.

**Royal College of Surgeons of England.**

The following gentlemen passed the First Professional Examination in Anatomy and Physiology for the Diploma of Fellow at a meeting of the Board of Examiners on May 18th: Alexander G. R. Foulerton, M.R.C.S., of St. Bartholomew's Hospital; Clement B. Voisey, M.R.C.S., of Owens College, Manchester, and St. Mary's Hospital; Frank N. Windsor, of Owens College, Manchester, and Cambridge University; Charles F. M. Ward, of Queen's College, Birmingham; Francis H. Marson, of Durham University, and Queen's College, Birmingham; Wilfred Edgcombe, of University College, Liverpool; James Musgrove, M.R.C.S., of Edinburgh University; William G. Laws, of Durham and Edinburgh Universities and St. Thomas's Hospital; George Lawrence, of Cambridge University and Guy's Hospital; and John Hartley, M.R.C.S., of Middlesex Hospital. (Eleven candidates were referred.)

### THE STUDENT OF MEDICINE—(*continued*).

Passed on the 19th inst.: Fancis H. Langlands, of Melbourne University and Middlesex Hospital; Frederick C. Scotson, James B. Carter, and John Stephenson, of Owens College, Manchester; Harold B. Dickinson, of University College, Liverpool; Charles W. Curtis, of Middlesex Hospital; Albert E. Martin, M.R.C.S., of Durham University and London Hospital; Arthur H. Cheatle, M.R.C.S., and William Turner, of King's College. (Eleven candidates were referred.)

Passed on the 22nd inst.: Edward Cotterell, M.R.C.S., of University College; Kenneth Lawson, of Middlesex Hospital; and Ernest W. Ormerod, of Cambridge University and St. Bartholomew's Hospital. (Eighteen candidates were referred.)

Passed on the 23rd inst.: John S. Sloane, Henry B. Maingay, and Henry S. Elworthy, of St. Bartholomew's Hospital; William F. Lucas and Francis E. Rock, of Middlesex Hospital; William J. Harris, of Cambridge University and Guy's Hospital; Charles S. Pantin, John B. Leathes, Richard H. Luce, and Daniel W. Samways, of Guy's Hospital. (Eleven candidates were referred.)

Passed on the 25th inst.: Henry A. D. Dickson, of St. Thomas's Hospital; Edward S. Cardell, of St. Bartholomew's Hospital; Alfred Foster, M.R.C.S., of St. Mary's Hospital; Arnold Chaning-Pearce and David M. Beddoe, of Guy's Hospital; Francis W. J. Cocker and George B. Robinson, of London Hospital. (Thirteen candidates were referred.)

103 candidates presented themselves for this examination, 39 of whom passed, and 64 referred for six months.

### Society of Apothecaries of London.

The following candidates passed in the subjects indicated during May:—

Surgery.—V. H. Barr. Guy's Hospital; H. B. Bates, University College, Liverpool; G. T. B. Blick, St. Mary's Hospital; T. F. Daniels, Royal Infirmary, Manchester; E. Middlebrooke, University of Iowa; H. E. Pittway, University College; W. K. Steele, Guy's Hospital; H. E. Tomlinson, Yorkshire College, Leeds; and L. Whitby, Royal Free Hospital.

Medicine, Forensic Medicine, and Midwifery.—F. G. Bullmore, St. Mary's Hospital; R. T. Cassall, University College; G. E. T. Haydon, London Hospital; R. M. Hughes, University College; W. J. C. B. Pitt, Queen's College, Birmingham; H. W. Roberts, St. George's Hospital; W. Robinson, Middlesex Hospital; H. B. Shepherd, Middlesex Hospital; H. E. Tomlinson, Yorkshire College, Leeds; E. B. Wrench, Cambridge University and St. Thomas's Hospital; W. Wright, St. Bartholomew's Hospital; and W. S. Wright, St. Mary's Hospital.

Medicine and Forensic Medicine.—T. F. Daniels, Owens College, Manchester, and R. A. Smith, Sheffield and Leeds.

Medicine and Midwifery.—J. F. Brown, Toronto University, and M. Jenkins, Guy's Hospital.

Midwifery and Forensic Medicine.—H. S. Cooper, Westminster Hospital.

Midwifery.—W. E. S. Jones, Guy's Hospital.

Forensic Medicine.—W. H. Savery, Sheffield.

To Messrs. Cooper, Haydon, Hughes, Middlebrooke, Preston, Shepherd, Tomlinson, Wrench, W. Wright, and W. S. Wright was granted the Diploma of the Society, qualifying for registration, and entitling them to practise Medicine, Surgery, and Midwifery.

### ATHLETICS.

King's College Sports (June 6th).—The twenty-fourth annual sports of this college were held on Saturday at Wormwood Scrubbs, in very successful style. For the third year in succession C. Wace won the mile challenge cup. Results:—Putting the Weight (16lb.): G. M. Hethrington, 28ft., 1; C. Brazil, 26ft. 11in., 2.—Throwing the Cricket Ball: E. W. Adams, 97yds. 3in., 1.; J. G. Hamilton, 86yds. 1ft. 4in., 2.—120 Yards Hurdle Race: Final Heat, Hethrington, 1; Balioziom, 2; Mawson, 3; Watson, 0; Hamilton, 0; Gooch, 0. Won by three yards; a yard between second and third. Time, 18 1·5sec.—Long Jump: G. M. Hethrington, 19ft. 4in., 1; C. Balioziom, 2; R. T. Tanner, 0.—High Jump: G. M. Hethrington, 5ft. 1in., 1; E. W. Adams, 5ft., 2; C. Balioziom, 0.—440 Yards Handicap (Boys of King's College School): F. Lonsdale, 10 yards start, 1; W. Wade, 8, 2; A. F. MacKinnon, 20, 3. Won by five yards. Time, 6 4·5 sec.—100 Yards Handicap: Final Heat, Hethrington, 1; Balioziom, 2; W. R. Smith, 3; Hamilton, 0; Holder, 0; Jones, 0. After a no race Hethrington won by a yard and a half; a yard separated second and third. Time, 10 3·5 sec.—One Mile Race (for Challenge Cup): C. Wace (holder), 1; G. W. A. Cooper, 2; W. A. Good, 3; G. W. T. Besive, 3. Wace had the pace of his men throughout, and won by sixty yards; forty between second and third men. Time, 4min. 47sec.—440 Yards Race: W. R. Smith, 1; G. G. Hamilton, 2; T. F. Jones, 3; E. C. Bailey, 0; O. H. Benson, 0; G. O. Pierce, 0; W. Dempster, 0; F. H. Jennings, 0; H. M. Jones, 0; C. Balioziom, 0. Won by five yards, two between second and third. Time, 58 4·5 sec. Half-Mile Handicap: C. Wace, scratch, 1; J. W. Cooper, 5 yards start, 2; N. S. Flores, 50, 3; H. Rogers, 35, 0; W. L. Buckland, 50, 0; W. H. Cannon, 55, 0; E. L. Rusby, 80, 0; G. Coad, 40, 0; A. Watson, 80, 0; F. A. Mackinnon, 80, 0; A. C. Bailey, 15, 0; J. O. Piarce, 10, 0. Won easily by thirty yards; five between second and third. Time, 2 min. 11 1·5 sec. 220 Yards Handicap: Final Heat: Halder, 1; Watson, 2; W. R. Smith, 3; Buckland, 0; Jones, 0; Cartwright, 0; Bailey, 0; Edwards, 0. Won by a yard; a foot between second and third. Time, 25 2·5 sec. Three Miles Handicap: C. Wace, scratch, 1; G. W. A. Cooper, scratch, 2; G. W. Biscoe, 400 yards start, 3; G. Coad, 350, 0; T. H. Bailey, 400, 0; W. A. Gooch, 100, 0; E. C. Bailey, 150, 0. Wace was leading at the commencement of the last mile, and he eventually won by 120 yards; a trifle less separated second and third. Time, 15 min. 42 sec. Obstacle Race: G. A. Mawson. Consolation Race: Jennings. Servants Race: Wright. Band Race: Grainger.

Resident Medical Superintendent.

